# May we routinely spare hippocampal region in primary central nervous system lymphoma during whole brain radiotherapy?

**DOI:** 10.1186/s13014-023-02251-2

**Published:** 2023-10-02

**Authors:** Ciro Mazzarella, Silvia Chiesa, Lucrezia Toppi, Stefan Hohaus, Simona Gaudino, Francesco D’Alo, Nicola Dinapoli, Resta Davide, Tiziano Zinicola, Serena Bracci, Antonella Martino, Francesco Beghella Bartoli, Elisabetta Lepre, Roberta Bertolini, Silvia Mariani, Cesare Colosimo, Vincenzo Frascino, Gian Carlo Mattiucci, Maria Antonietta Gambacorta, Vincenzo Valentini, Mario Balducci

**Affiliations:** 1 UOC di Radioterapia Oncologica, Dipartimento Diagnostica per Immagini, Radioterapia Oncologica ed Ematologia, Fondazione Policlinico Universitario “A. Gemelli” IRCCS, largo A. Gemelli, Rome, Italy; 2 UOC di Ematologia, Dipartimento Diagnostica per Immagini, Radioterapia Oncologica ed Ematologia, Fondazione Policlinico Universitario “A. Gemelli” IRCCS, Rome, Italy; 3 Istituto di Ematologia, Università Cattolica del Sacro Cuore, Rome, Italy; 4 UOC di Neuroradiologia, Dipartimento Diagnostica per Immagini, Radioterapia Oncologica ed Ematologia, Fondazione Policlinico Universitario “A. Gemelli” IRCCS, Rome, Italy; 5 Istituto di Radiologia, Università Cattolica del Sacro Cuore, Rome, Italy

**Keywords:** PCNSL, Hippocampal sparing, WBRT, IMRT, Neuro-toxicity, Personalized treatment

## Abstract

**Purpose:**

One of the main limiting factors of whole-brain radiation therapy (WBRT) for primary central nervous system lymphoma (PCNSL) is the impairment of neurocognitive functions (NCFs), which is mainly caused by radiation-induced injury to the hippocampus. With a view to preventing NCF impairment and personalizing treatment, we explored the feasibility of sparing the hippocampus during WBRT by correlating the sites of PCNSL lesions with the hippocampus.

**Methods and materials:**

Pre-treatment MR images from patients who underwent WBRT between 2010 and January 2020—and post-radiotherapy images in cases of relapse—were imported into the Varian Eclipse treatment-planning system and registered with the simulation CT. We constructed three 3-dimensional envelopes around the hippocampus at distances of 5, 10 and 15 mm and also contoured primary lesions and recurrences.

**Results:**

We analyzed 43 patients with 66 primary lesions: 9/66 (13.6%) involved the hippocampus and 11/66 (16.7%) were located within 5 mm of it. Thirty-six lesions (54.5%) were situated more than 15 mm from the hippocampus, while 10/66 (15.2%) were between 5 and 15 mm from it. The most common location was in deep brain structures (31%). Thirty-five of the 66 lesions relapsed: in field in 14/35 (40%) and outfield in 21/35 (60%) in different sites. Globally, 16/35 recurrences (45.7%) were located in the hippocampus or within 5 mm of it.

**Conclusion:**

These data show that routinely sparing the hippocampus is not feasible. This approach could be considered in selected patients, when the lesion is more than 15 mm from the hippocampus.

## Introduction

Primary central nervous system lymphoma (PCNSL) is a rare form of aggressive extranodal non-Hodgkin’s lymphoma (NHL) [1], accounting for 1–2% of all non-Hodgkin’s lymphomas and 2–3% of all primary brain tumors [2]. PCNSL is an aggressive tumor; it has a rapid clinical evolution and a rather unfavorable prognosis, with 5-year survival rates of 30 to 50% and a median overall survival of 42 months (range 36–60 months) [3].

Owing to its rarity, it has been difficult to carry out randomized studies aimed at defining a therapeutic standard. Surgery is restricted to biopsy, while chemotherapy and radiotherapy are the main therapeutic approaches [4, 5]. Currently, the preferred regimen for PCNSL involves chemotherapy followed by consolidation treatments. Chemotherapy regimens designed to penetrate the blood-brain barrier, mainly based on high-dose methotrexate (HD-MTX) [6] in combination with other agents [3] and subsequent whole-brain radiotherapy (WBRT) or high-dose chemotherapy with stem cell rescue (HDC-ASCT), are considered the standard of therapy. WBRT is generally undertaken. This approach is justified by the fact that intracranial lesions are frequently multicentric and microscopic infiltration is common [7, 8]. Indeed, brain relapses increase when the treatment volume is reduced, as reported by Shibamoto [8]. Relapse and/or progression of the disease are the main causes of treatment failure, while late neurotoxicity is the main limiting factor [4, 9–12]. The mechanisms underlying the development of neurotoxicity are only partly understood and are related in part to the neoplasm and in part to the effects of treatment. Recent studies have suggested that, in patients undergoing panencephalic radiotherapy, deficits in both short-term and long-term memory and in temporospatial perception are related to hippocampal injury [13, 14]. The pathogenesis of radio-induced neurocognitive impairment could be due, at least in part, to a microangiopathy that causes infarcts and microvascular ischemia of the critical centers of neurocognitive processes, especially in the hippocampus [15]. In addition to its fundamental role in learning and memory processes, the hippocampus has recently been suggested to act as a source of stem cells involved in neuro-regeneration [16]. In this context, hippocampal sparing has been explored in patients affected by brain metastases who undergo WBRT [13, 14, 17, 18]. Other studies have investigated the role in neurocognitive functions of other sites, such as white matter [19]; unfortunately, owing to its ubiquitous anatomical location in the brain, the white matter cannot be avoided during panencephalic radiotherapy.

In this study, we describe the distribution of PCNSL lesions within the brain, both on diagnosis and at the time of recurrence, in relation to the hippocampus, in order to evaluate the feasibility of hippocampal sparing during WBRT with a view to reducing neurocognitive impairment and personalizing radiation treatment.

## Methods and materials

This is a monoinstitutional retrospective study. Adult patients (≥ 18 years of age) affected by PCNSL with exclusively intracranial disease were eligible. The eligibility criteria for radiation therapy were: ECOG Performance Status ≤ 4, pathologically proven diagnosis of PCNSL and follow-up of at least one year to evaluate the site of any recurrences in relation to the hippocampus. Exclusion criteria were: positive HIV serology or presence of immunodeficiency syndromes, degenerative neurological or neuropsychiatric pathology at the time of diagnosis of PCNSL, pregnancy and heart, respiratory or kidney failure. The study received ethics approval from the ethical committee of our institute.

**Fig. 1:**
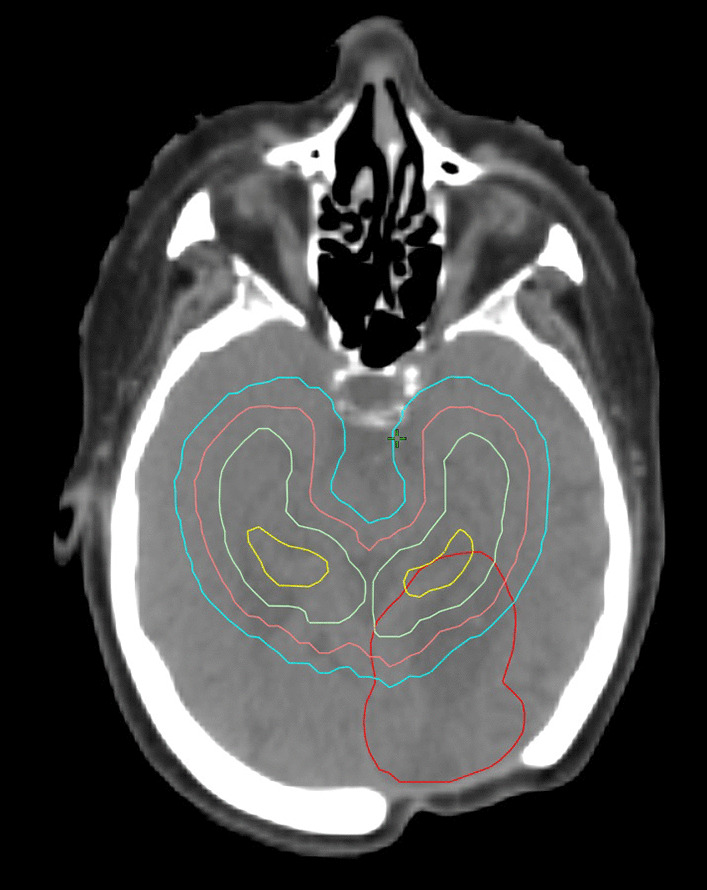
Contouring of hippocampus with 5-, 10-, and 15-mm expansion envelopes and lesion. (Yellow lines = hippocampus – Green lines = 5 mm envelope – Pink lines = 10 mm envelope – Blue lines = 15 mm envelope – Red lines = lesion)

In order to evaluate the spatial distribution of PCNSL lesions in relation to the hippocampus, we retrospectively analyzed the simulation CT scans and MRI data-set ofpatients who had undergone WBRT from 2010 to January 2020. Brain MRI images before radiotherapy, and post-treatment in cases of relapse, were imported into the Varian Eclipse treatment-planning system and co-registered with the simulation CT for contouring. T1-weighted, post-contrast, axial images were used to outline the hippocampus and PCNLS lesions manually. The hippocampus was contoured according to the atlas of neuroanatomy and to guidelines on T1-weighted MRI axial sequences [20, 21]. We contoured lesions, both on diagnosis and at the time of disease relapse; all contours were discussed with a neuroradiologist. Three 3-dimensional envelopes surrounding the hippocampus at distances of 5, 10 and 15 mm were created, in accordance with Amol Ghia’s 2007 study [14] (Fig. 1). Each lesion was assigned to a given range of distance from the hippocampus (< 5 mm, 5–15 mm, > 15 mm) or classified as situated inside it. Moreover, its location within the brain parenchyma was recorded, so that each lesion fell into a specific category for statistical analysis.

## Statistical analysis

Qualitative variables were expressed as absolute frequencies and percentages. The distances of the lesions from the hippocampus were described by means of descriptive statistics. Inclusion of the lesions within the pre-defined margins around the hippocampus was evaluated by means of McNemar’s chi-square test and χ^2^ for paired samples (pre- and post-treatment).

## Results

We examined the post-contrast T1-weighted MRI scans of 43 patients, performed before radiotherapy and on relapse, for a total of 66 primary lesions and 35 recurrences.

**Fig. 2:**
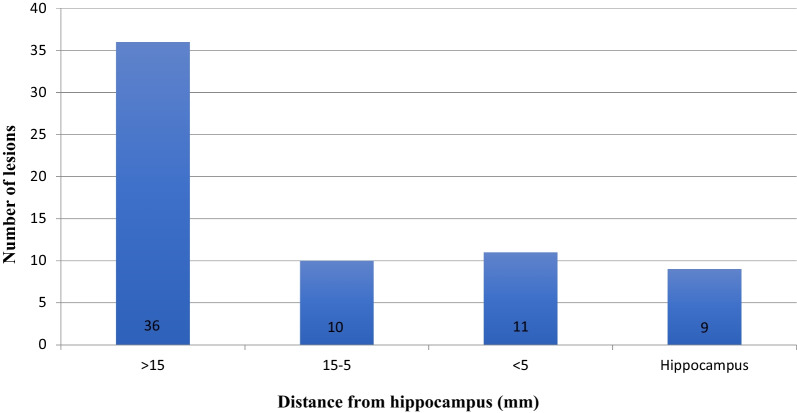
Location of lesions on diagnosis as a function of distance from the hippocampus

In the subset of primary lesions, we recorded the site of PCNLS lesions within the brain parenchyma; the most common location was deep brain (31.0%, n = 21) followed by parietal and frontal lobes (each site 20.0%, n = 13), temporal lobe (11.0%, n = 7), cerebellum, occipital lobe and orbital region (each 6.0%, n = 4) (Table 1). We observed that: 11/66 lesions (16.7%) were located less than 5 mm from the hippocampus, 9/66 (13.6%) involved the hippocampus, 36/66 lesions (54.5%) were more than 15 mm, and 10 (15.2%) were 5–15 mm from the hippocampus (Fig. 2). In the subgroup of lesions localized in the hippocampus and less than 5 mm from it, 13/20 (65.0%) were in the deep brain, 2/20 (10.0%) in the parietal lobe, 2/20 in the occipital lobe, 2/20 (10.0%) in the cerebellum and 1/20 (5.0%) in the temporal lobe. Of the 36 lesions localized more than 15 mm from the hippocampus, 11 (30.5%) were in the frontal lobe, 9 in the parietal lobe (25.0%) 5 in the deep brain (13.9%), 4 in the temporal lobe (11.1%), 4 in the orbital region (11.1%), 2 in the occipital lobe (5.6%) and only 1 (2.8%) in the cerebellum. Regarding lesions localized between 5 and 15 mm from the hippocampus, 3 (30%) were localized in the deep brain, 2 (20%) in the frontal lobe, 2 (20%) in the parietal lobe, 2 (20%) in the cerebellum, and only 1 (10%) in the temporal lobe.

**Fig. 3:**
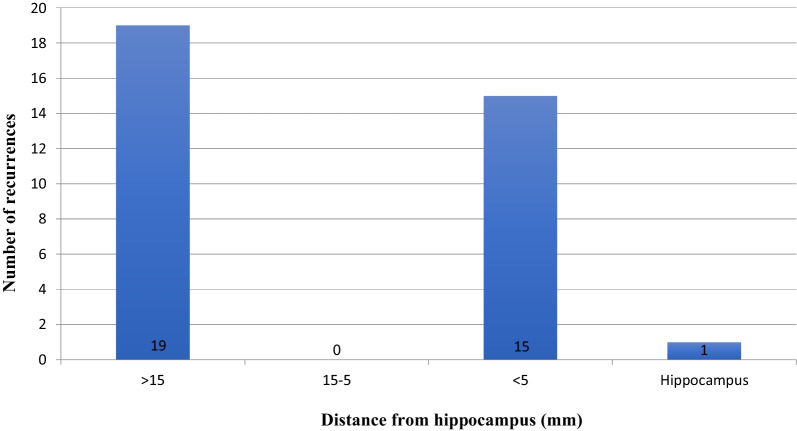
Location of recurrences as a function of distance from the hippocampus

With regard to the 35 recurrences, 12/35 (34.3%) involved the deep brain, 9/35 (25.7%) the frontal lobe, 4/35 (11.4%) the parietal lobe, 3/35 (8.5%) the occipital lobe, 3/35 (8.5%) the cerebellum, 1/35 (2.9%) the temporal lobe, 1/35 (2.9%) the orbital region, 1/35 (2.9%) the spinal cord and 1/35 (2.9%) the hippocampus. Fourteen of the 35 (40%) relapses occurred in the same location as the primary lesions; 6/14 (42.8%) of these were in the hippocampus or less than 5 mm from it. Twenty-one out of 35 (60%) recurrences were observed in sites different from that of the primary lesion; 10/21 (47.6%) of these were near to the hippocampus. Thus, 16/35 (45.7%) recurrences were observed in the hippocampal region or less than 5 mm from it (Fig. 3). We observed that 4/36 lesions (11.1%) located more than 15 mm from the hippocampus on diagnosis relapsed at a distance of less than 5 mm from the hippocampus, and none relapsed in the hippocampus. The McNemar test showed no correlation between the pre-treatment and post-treatment locations of the lesions, on considering a threshold distance of 5 mm from the hippocampus (*p* value 0.55). The overall rate of response to treatment was evaluated by means of the McNemar test, which showed a *p* value = 0.001 (the null hypothesis was no difference between the number of lesions before and after treatment). A similar pattern emerged in the different sub-groups (> 15 mm: *p* value = 0.012; 15 mm to 5 mm: *p* value = 0.016; <5  mm: *p* value = 0.52; hippocampal lesion: *p* value = 0.013).

**Table 1:** Locations of lesions on diagnosis

Location	N°	%
Deep brain	21	31.0
Parietal lobe	13	20.0
Frontal lobe	13	20.0
Temporal lobe	7	11.0
Occipital lobe	4	6.0
Cerebellum	4	6.0
Orbital region	4	6.0
Total	66	100.0

## Discussion

In this study, we analyzed the sites of PCNSL lesions in relation to the hippocampus in order to evaluate the possibility of sparing the hippocampal region, so as to prevent neurocognitive impairment and personalize treatments. Over the past several decades, it has been established that the hippocampus plays an essential role in memory function [22]. Much evidence has suggested that the pathogenesis of radiation-induced NCF deficit involves radiation-induced injury to proliferating neuronal progenitor cells in the subgranular zone of the hippocampus [23, 24]. Small doses of radiation cause apoptosis in the subgranular zone, whereas little or no apoptosis is observed in other areas of the cerebrum [22, 25]. Modern intensity-modulated radiotherapy (IMRT) techniques have therefore been developed to avoid conformally the hippocampal neural stem-cell niche during WBRT, in order to try to reduce neurocognitive deficit after WBRT. Indeed, IMRT techniques enable the hippocampus to be spared while achieving acceptable target coverage and homogeneity [26]. On the basis of this evidence, several studies have been carried out in order to assess the feasibility of sparing the hippocampal volume. In 2007, Amol Ghia et al. [14] found that only 3% of 270 metastases were located in proximity to the hippocampus, thus leading to the conclusion that the hippocampal volume could be spared. An additional study was conducted by Gondi in 2010; this showed that 91% of newly diagnosed patients with brain metastases were eligible for hippocampal sparing (HS) WBRT [20]. The role of HS in reducing NCF deficit during panencephalic radiotherapy has been demonstrated in several recent trials, such as the RTOG 0933 trial [26, 27]. These trials have shown that HS WBRT has fewer adverse neurocognitive effects than WBRT alone. In addition, in the recently published phase III study by Paul D. Brown et al., sparing the hippocampal region in the panencephalic treatment of patients with brain metastases proved significantly beneficial in terms of preserving neurocognitive functions [14] Is well documented as the WBRT is the standard target volume in patients with PCNSL.

However the WBRT is related to a neurocognitive danage. For this reason, different studies explored low-dose WBRT strategies in association with intensified chemotherapy regimens. A multicentre phase II study investigated the efficacy of rituximab, procarbazina and vincristine followed by consolidation reduced-dose whole-brain radiotherapy (23.4) and cytarabine after complete response versus standard WBRT ( 45 Gy). The 2-years PFS for the first group was 77% with less neurotixicity and a better cognitive performace [28].

Given radiotherapy neurocognitive toxicity-related, we wondered if the hippocampus could also be spared in this setting.

To our knowledge, the present study is the first in which the sites of PCNSL lesions have been analyzed with a view to evaluating the possibility of routinely sparing the hippocampal region during whole-brain radiotherapy, in order to preserve NCFs and enable personalized treatment. We observed that, at the time of diagnosis, 30% of lesions either involved the hippocampus or were located within 5 mm of it. Moreover, when we analyzed the sites of disease recurrence, we observed that 14 lesions (40%) recurred at the site of the primary tumor, while 21 (60%) were in different locations. Globally, 16/35 (45.7%) recurrences were observed in the hippocampal region or less than 5 mm from it; 4/36 lesions (11.1%) that were located more than 15 mm from the hippocampus at the time of diagnosis relapsed at a distance of less 5 mm from it, but none of these relapsed in the hippocampus. Considering that only one of the lesions located in the hippocampal region at the time of diagnosis recurred in the hippocampus and that, on diagnosis, 30% of lesions were situated in the hippocampus or within 5 mm of it, we can conclude that routine hippocampal sparing is not feasible. We could, however, hypothesize sparing the hippocampal region if the lesion is located more than 15 mm from the hippocampus. The clinical incidence of hippocampal or perhippocampal lesions after conformal-avoidance WBRT n.eeds to be elucidated in a prospective clinical trial, in order to clarify the benefits and risks of hippocampal sparing during WBRT in selected cases. We also recorded the location of PCNSL lesions within the brain parenchyma, and found that the most common sites of both primary lesions and relapses were in the deep brain; this finding is at variance with the literature reports.

## Conclusions

Our study does not support the routine sparing of the hippocampal region; however, this approach could be considered in selected patients, when the PCNSL is more than 15 mm from the hippocampus.

## Data Availability

All supporting data is available.

## Abbreviations

WBRT: Whole-brain radiation therapy
PCNSL: Primary central nervous system lymphoma
NCFs: Neurocognitive functions
NHL: Non-Hodgkin’s lymphoma
IMRT: Modern intensity-modulated radiotherapy
HS: Hippocampal sparing
